# Type D Personality and Health-Related Quality of Life in Vascular Surgery Patients

**DOI:** 10.1007/s12529-018-09762-3

**Published:** 2019-07-01

**Authors:** Elke Bouwens, Felix van Lier, Ellen V. Rouwet, Hence J. M. Verhagen, Robert Jan Stolker, Sanne E. Hoeks

**Affiliations:** 1 Department of Anesthesiology, Erasmus MC University Medical Center Rotterdam, Erasmus MC, NA-1718, P.O. Box 2040, 3000 CA Rotterdam, Netherlands; 2 Department of Vascular Surgery, Erasmus MC University Medical Center Rotterdam, Rotterdam, Netherlands

**Keywords:** Patient-reported outcomes, Quality of life, Type D personality, Vascular disease

## Abstract

**Background:**

This study evaluated the association of type D personality and health-related quality of life (HRQoL) and assessed the stability of type D personality in vascular surgery patients during the year after surgery.

**Method:**

In a prospective cohort study between 2008 and 2014, 294 patients were assessed with validated questionnaires preoperatively and at 1, 6, and 12 months after surgery. Associations between type D personality, depression, and HRQoL were analyzed by generalized estimating equation models. Type D personality was analyzed in its standard dichotomous form as well as continuous (*z*) scores of its two components, negative affectivity (NA) and social inhibition (SI), and their interaction term.

**Results:**

Prevalence of type D personality varied between 18% and 25%. However, only 9% of the complete responders were classified as type D personality at all four assessments, whereas one third changed between type D classifications. Continuous scores showed greater stability over time. Dichotomized type D personality measured over time was significantly associated with impaired HRQoL, but this was not the case if measured once at baseline, like in general use. The continuous NA score and depression were also significantly associated with impaired HRQoL over time.

**Conclusion:**

Type D personality was not a stable trait over time. Preoperative assessment of type D personality did not predict improvement in HRQoL after vascular surgery. However, the study revealed associations between the NA component of type D personality, depression, and lower HRQoL. This indicates that measures of overall negative affect should be taken into account when assessing HRQoL patient-reported outcomes in vascular surgery patients.

## Introduction

Surgical outcomes have traditionally focused on treatment success, morbidity, and mortality. In recent years, however, emphasis on health-related quality of life (HRQoL) is gaining momentum. HRQoL is a multidimensional concept that encompasses the physical, emotional, and social components associated with an illness or its treatment as perceived by the patient. Patients with peripheral artery disease or with aortic aneurysm disease, experience a diminished HRQoL due to walking disability, a physically inactive lifestyle, and emotional stress [1–5]. Consequently, the goal of treatment for vascular patients is not limited to relieving symptoms and prolonging life, but foremost to improve HRQoL [3, 6]. Since the effectiveness of cardiovascular care is increasingly being evaluated from patients’ perspectives using HRQoL questionnaires [7], it is essential to determine risk factors that could predict poor HRQoL in vascular surgery patients.

Per definition, HRQoL is a patient-reported outcome which assesses patients’ perceived lifestyle impact of their disease and its treatments [7]. Differences in patient-reported health status, however, do not necessarily concur with the patient’s objective clinical status or with the physician’s evaluation of the patient’s health. Rather, psychological and social factors may also impact a patient’s perception of his or her health. The distressed (type D) personality includes a personality trait characterized by negative affectivity (NA) and social inhibition (SI). Patients with this personality trait are likely to experience increased levels of anxiety and depressed mood across situations and time, while not sharing these emotions with others [8].

Type D personality has been shown to correlate with impaired patient-reported physical and mental health status as well as increased mortality in various cardiovascular patient groups, independent of demographic and clinical characteristics and baseline health status [9–13]. A meta-analysis concerning cardiovascular patients showed that type D was associated with twofold increased odds of impaired physical health status in 3035 patients (odds ratio 1.94, 95% confidence interval (CI) 1.49–2.52) [9]. Type D personality was also found to be a strong predictor of disease impact in patients with intermittent claudication [14]. Furthermore, type D personality is mentioned in the current European Cardiovascular Prevention guideline as one of the psychological risk factors, which may impede lifestyle changes or adherence to treatment in cardiovascular patients [15]. Consequently, it may be crucial to take the patient’s personality profile into account when evaluating HRQoL, particularly if this is used to assess the effectiveness of (surgical) treatments.

A meta-analysis published in 2012 supported the overall finding of an association between type D personality and prognosis in 5341 patients with cardiovascular disease [16]. Interestingly, however, they point out that high-quality methodological studies failed to confirm the association and that the strength of the association declined over the years, suggesting that early studies about type D personality had overestimated the prognostic importance [16]. Concerning the type D personality construct, both dimensions (NA and SI) are dichotomized by median splits, resulting in four different possible categories. Most studies reduce this to a dichotomous classification of a type D personality or a non-type D personality [17]. The typical dichotomous approach of type D measurement, however, has been criticized in recent literature and raised the question whether type D personality is really categorical and able to differentiate between true cases and non-cases [18]. This directs the importance of including continuous scores of NA, SI, and their interaction term NA by SI in research analysis [18, 19]. Furthermore, since the stability of type D personality seems to differ over time in patients with cardiovascular disease [20–22], the question arises whether one (baseline) measurement of type D personality is sufficient.

The aim of the present study was to evaluate the association between type D personality and HRQoL in patients undergoing vascular surgery during the first postoperative year and to assess the stability of type D personality, including its continuous NA and SI scores and their interaction term.

## Methods

### Patients and Design

The current study was based on a prospective, observational, single-center cohort study, with a 12-month follow-up. All patients referred to the anesthesiology outpatient clinic prior to elective vascular surgery at a tertiary medical center in the Netherlands were considered eligible study subjects. Patients with a life expectancy less than 12 months; presence of severe psychopathological comorbidities (e.g., psychosis, suicidal ideation); or insufficient knowledge of the Dutch language were excluded from participation in the study. A total of 481 unique patients were included in the cohort at the outpatient clinic between September 2008 and June 2014. All included patients were requested to fill out validated questionnaires for HRQoL, type D personality, and depression at four different time points: before surgery and 1, 6, and 12 months after surgery.

Elective vascular procedures were divided into aortic surgery, carotid endarterectomy, and lower limb arterial reconstruction. Aortic surgery included endovascular or open repair of abdominal or thoracic aneurysms, aortic bifurcation prostheses for aortoiliac occlusive disease, and removal of infected aortic grafts. Lower limb arterial procedures included peripheral bypass surgery, common femoral endarterectomy, removal of infected peripheral grafts, and (false) peripheral aneurysm repair.

Following screening, 187/481 patients eligible for vascular surgery (39%) were excluded for the current analysis: 48 did not undergo surgery, 29 had cognitive impairment (Mini Mental Status Examination (MMSE) score of 23 or lower), 17 had a missing MMSE score, and 93 cases had missing data in the type D personality questionnaire at baseline. A total number of 294 patients were used for the current analysis.

This study was approved by the medical ethics committee of the medical center (NL24093.078.08.) and informed consent was obtained from all individuals included in the study.

### Type D Personality

The type D Scale 14 (DS14) was used to assess type D personality. The DS14 is a validated [8] and widely used questionnaire [9–12, 20, 22–26] consisting of 14 items with a 5-point Likert scale ranging from 0 (false) to 4 (true). The questionnaire measures both negative affectivity (NA) and social inhibition (SI). NA refers to the propensity to experience negative emotions across time and situations, while SI refers to the propensity to inhibit the expression of emotions in social interactions. Patients are classified as Type D personality if they have a score of 10 or more on both subscales [27]. Furthermore, regarding the ongoing discussion about this typical dichotomous approach and the well-established statistical drawbacks of forced dichotomization [28], both the dichotomous form of type D personality and the continuous scores of NA, SI, and their interaction term NA by SI were analyzed. The type D personality hypothesis is fundamentally about the synergistic interaction of NA and SI, which should be tested as the product of continuous NA and SI scores, while controlling their first-order effects [19].

### Health-Related Quality of Life

One of the most frequently applied health status measures is the EuroQol 5 dimensions questionnaire (EQ-5D) [29, 30]. The EQ-5D imposes minimal burden as it is a brief, simple questionnaire for patients to understand and to complete [31]. The Dutch version was used as standardized, generic instrument for describing and valuing health. The EQ-5D-3L consists of a descriptive system that defines health along five dimensions (mobility, self-care, usual activities, pain/discomfort, and anxiety/depression), each with three response options. The calculated EQ-5D index represents the patient’s self-rated health, where scores < 0 are regarded as worse than death and 1 is representing full health, from the perspective of the general population.

### Depression

Self-reported depressive symptoms were assessed with Patient Health Questionnaire-9 (PHQ-9), an abbreviated version of the Primary Care Evaluation of Mental Disorders questionnaire [32]. The PHQ-9 is designed as a screening tool and quantifies the frequency of DSM-IV criteria. Items are answered on a 4-point Likert scale; scores are summed to create a score between 0 and 27 and are then dichotomized by a cutoff score of ≥ 10 to indicate at least moderate depression. With this cutoff point, the PHQ-9 has a sensitivity and specificity of 88% for major depression [33]. Additionally, two recent meta-analyses have shown good pooled sensitivity (0.80 and 0.77, respectively) and specificity (0.92 and 0.94, respectively) for PHQ-9 against the DSM-IV diagnosis of major depressive disorder or major depressive episode in the settings of primary care clinics and clinics (other than psychiatric clinics) [34, 35].

### Statistical Analyses

For baseline characteristics, patients were categorized into a type D (i.e., high negative affectivity and social inhibition) or a non-type D personality group at baseline according to the DS14. Baseline variables with a normal distribution are presented as mean ± standard deviation, whereas the median (interquartile range) are presented in case of non-normality. Categorical variables are presented as counts (percentages). Differences between patients with a type D personality and a non-type D personality for categorical data were compared using the Chi-square test (Fisher’s exact tests when appropriate), and continuous variables with Student’s *t* test as parametric test or Mann-Whitney *U* test as non-parametric test.

Internal consistency was evaluated using Cronbach’s *α* coefficient, which refers to the extent to which the items within a scale are inter-related. Cronbach’s *α* coefficient > 0.7 are generally regarded as acceptable for psychometric scales, although > 0.9 is recommended for individual patient assessment [36]. Prevalence and validity in terms of stability over time in type D personality were analyzed for all participants available at each time point (i.e., baseline to 1 month, 6 months, and 12 months). The stability between different time points of the dichotomized type D personality and its dichotomized subscales NA and SI were examined by Cohen’s *κ*. Intraclass correlation coefficients (ICC) were assessed for stability of the continuous scores NA, SI, and the interaction of NA by SI.

The change in HRQoL over time and its association with type D personality were analyzed by regression models using generalized estimating equation (GEE) with an exchangeable correlation structure. GEE is a suitable method to analyze repeated measures within an individual and can accommodate missing values [37]. In all GEE models, HRQoL measured with the EQ-5D index was the dependent outcome variable and time was included in all models as a categorical variable, represented by three dummy variables to investigate the development of HRQoL over time (i.e., baseline to 1 month, 6 months, and 12 months). Interaction terms were tested and included in models if *p* value was less than .10.

In view of the ongoing debate concerning the dichotomization of two continuous scales into one typology [18, 19], we also analyzed the *z* scores of the continuous components NA, SI, and their interaction term NA by SI. To this end, the GEE models were separately fitted for dichotomized type D personality as well as for the continuous *z* scores of NA, SI, and the interaction term NA by SI (while controlling their first-order effects). Dichotomized type D personality and the continuous scores were included as time-dependent covariates, i.e., allowing values to change over the different time points. Multivariable analyses were performed for these GEE models, including time-dependent depression, age, gender, and marital status. Heart failure, chronic obstructive pulmonary disease, and diabetes were also included in these multivariate models due to its known association with HRQoL [38–40].

Generally, the presence of type D personality is assessed only once, e.g., preoperatively. To determine whether a single baseline type D measurement is predictive for postoperative HRQoL, we repeated foregoing GEE models with baseline dichotomized type D personality and with the baseline continuous *z* scores of NA, SI, and NA by SI.

A two-sided *p* value < .05 was considered statistically significant for all tests. All analyses were performed with IBM SPSS Statistics Version 24.0 (Armonk, NY: IBM Corp.).

## Results

### Study Population

A total of 294 patients were included in the study. Excluded patients were more often women (32% vs. 18%, *p* = <.001), were more frequently single (i.e., living alone, divorced, widow, or widower) (42% vs. 26%, *p* < .001) and had a lower BMI (25 vs. 26, *p* = .001). No other differences in demographic characteristics or clinical factors were found. Per definition, all included patients (82% male, age 69 ± 9 years) completed the questionnaire at baseline. Of these participants, 244 responded to the questionnaire 1 month after surgery (85% response rate), 221 after 6 months (79% response rate), and 217 patients after 12 months (78% response rate); 165 patients (56%) responded at all follow-up moments. Incomplete responders were more often women (*p* = .039), singles (*p* = .028), and smokers (*p* = .010), but no other differences in demographic characteristics or clinical factors were found.

### Type D Personality over Time

**Fig. 1:**
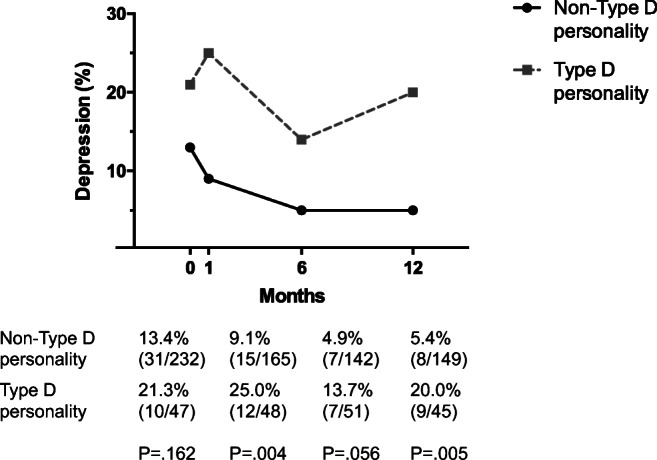
Percentage of patients with depression determined by the PHQ-9 questionnaire at baseline (0) and at 1, 6, and 12 months follow-up, stratified by type D personality. PHQ-9 = 9-question Patient Health Questionnaire

The prevalence of type D personality was 18% before surgery (53/294), 23% at 1 month (54/234), 25% at 6 months (53/210), and 23% at 12 months after surgery (47/205). There were no significant differences in patient characteristics between patients with or without type D personality at baseline, except for age (66 years vs. 70 years, *p* = .001; Table 1). Overall, type D personality patients showed more depression on the PHQ-9 (Fig. 1).

**Table 1:** Baseline characteristics before surgery stratified by type D personality

Characteristics	Type D *n* = 53 18.0%	Non-Type D *n* = 241 82.0%	*p* value
Demographics			
Age	65.8 ± 9.8	70.2 ± 8.7	0.001
Gender (male)	44 (83.0)	197 (81.7)	0.83
Body mass index (kg/m^2^)	26.6 ± 3.8	26.8 ± 3.8	0.73
MMSE	28.0 (27.0–29.0)	28.0 (27.0–29.0)	0.80
Marital status (single)	17 (32.1)	60 (24.9)	0.28
Clinical factors			
Heart failure	5 (9.4)	26 (10.8)	0.77
Diabetes mellitus	10 (18.9)	46 (19.1)	0.97
Chronic obstructive pulmonary disease	14 (26.4)	58 (24.1)	0.72
Renal insufficiency	9 (17.0)	25 (10.4)	0.17
Hypertension	39 (73.6)	168 (69.7)	0.58
Smoking	18 (34.6)	82 (34.0)	0.94
Vascular procedure			0.38
Carotid endarterectomy	3 (5.7)	20 (8.3)	
Aortic surgery	28 (52.8)	144 (59.8)	
Lower limb arterial reconstruction	22 (41.5)	77 (32.0)	

Baseline variables with a normal distribution are presented as mean ± standard deviation, whereas the median (interquartile range) are presented in case of non-normality. Categorical variables are presented as counts (percentages)

Although the prevalence rates of about 20% could suggest stability in type D personality over time, Table 2 shows differently. As shown, only 67% of the type D personality patients at baseline were correspondingly diagnosed with a type D personality 1 month after surgery. Of the 165 participants that responded at all four time points, only 9% of the patients (14/165) were classified as a type D personality at each assessments, and only 56% (92/165) had a stable non-type D personality; 35% (59/165) of the patients changed between personality classifications. The prevalence of type D personality at baseline did not differ significantly between complete responders and those who did not complete one or more follow-ups (21% vs. 15%, *p* = .19).

**Table 2:** Changes over time in type D personality

		Preoperative			1 month after surgery		
		Type D personality	Non-type D personality	*p* value	Type D personality	Non-type D personality	*p* value
1 month after surgery	Type D personality	30 (66.7%)	24 (12.7%)	< .001			
	Non-type D personality	15 (33.3%)	165 (87.3%)				
12 months after surgery	Type D personality	20 (50.0%)	27 (16.4%)	< .001	21 (51.2%)	20 (14.1%)	< .001
	Non-type D personality	20 (50.0%)	138 (83.6%)		20 (48.8%)	122 (85.9%)	

Change over time in type D personality was analyzed for all participants available at each time point, resulting in 60 missing cases between baseline and 1 month after surgery, 89 missing cases between baseline and 12 months after surgery, and 111 missing cases between 1 and 12 months after surgery

Cronbach’s *α* indicated good internal consistency of the continuous DS14 at the individual time points (range 0.82 to 0.90). The highest values occurred at the follow-up moments (Table 3). In agreement to the shift observed between whether or not a type D personality, Cohen’s *κ* showed low stability of the dichotomized type D personality over time. The Cohen’s *κ* values for the dichotomized type D were fair to moderate (range 0.32 to 0.50), with the lowest values between baseline and 12 months after surgery (Cohen’s *κ* = 0.32). The dichotomized NA and SI subscales showed higher values (Table 4). Looking at the consistency over time for the continuous scores, the intraclass correlation coefficients were substantial to almost perfect. The highest intraclass correlation coefficients were observed between pre-surgery and 1 month after surgery (ICC 0.83 to 0.86) (Table 4).

**Table 3:** Cronbach’s *α*

	Preoperative (baseline)		1 month after surgery		6 months after surgery		12 months after surgery	
Continuous interaction term NA by SI	*α* = 0.83	*n* = 294	*α* = 0.89	*n* = 234	*α* = 0.89	*n* = 210	*α* = 0.90	*n* = 205
Continuous NA	*α* = 0.86	*n* = 294	*α* = 0.90	*n* = 238	*α* = 0.89	*n* = 216	*α* = 0.88	*n* = 209
Continuous SI	*α* = 0.82	*n* = 294	*α* = 0.84	*n* = 238	*α* = 0.84	*n* = 225	*α* = 0.87	*n* = 212

*α* Cronbach’s alpha, *n* number, *NA* negative affectivity, *SI* social inhibition

**Table 4:** Cohen’s *κ* and intraclass correlation coefficients

	Preoperative to 1 month after surgery			Preoperative to 12 months after surgery			1 to 12 months after surgery		
Cohen’s κ									
	*n*	*κ*	*p* value	*n*	*κ*	*p* value	*n*	*κ*	*p* value
Type D dichotomized	234	0.50	< 0.001	205	0.32	< 0.001	183	0.37	< 0.001
NA dichotomized	238	0.55	< 0.001	209	0.49	< 0.001	189	0.45	< 0.001
SI dichotomized	238	0.57	< 0.001	212	0.54	< 0.001	191	0.57	< 0.001
ICC									
	*n*	ICC	95% CI	*n*	ICC	95% CI	*n*	ICC	95% CI
Continuous interaction term NA by SI	234	0.87	0.837, 0.903	206	0.72	0.633, 0.788	184	0.74	0.646, 0.802
Continuous NA	238	0.83	0.776, 0.865	209	0.71	0.617, 0.778	189	0.75	0.661, 0.809
Continuous SI	238	0.86	0.813, 0.888	212	0.80	0.737, 0.847	191	0.83	0.774, 0.872

Cohen’s *κ* and ICC were analyzed for all participants available at each time point. When analyzing the 165 complete responders, both Cohen’s κ (0.37 to 0.61) and ICC (0.71 to 0.90) values increased slightly

*CI* confidence interval, *ICC* intraclass correlation coefficients, *n* number, *NA* negative affectivity, *SI* social inhibition

### Type D Personality and HRQoL

**Fig. 2:**
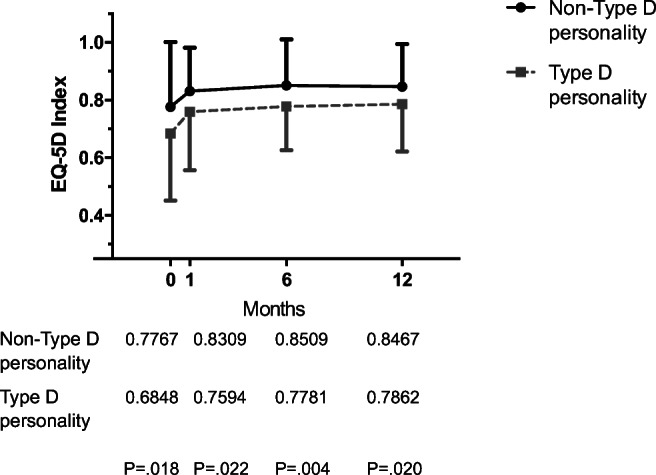
EQ-5D Index. Change in health-related quality of life over time at baseline (0) and at 1, 6, and 12 months follow-up, stratified by type D personality. Bars = standard deviations. EQ-5D = Euroqol 5 dimensions questionnaire

There was a significant overall improvement in HRQoL over time after vascular surgery according to the EQ-5D, as seen in Fig. 2 and in the regression coefficients at postoperative time points compared to baseline in Table 5. The main improvement in HRQoL was particularly observed 1 month after surgery; summary index scores stabilized thereafter (Fig. 2).

**Table 5:** Generalized estimating equation models to predict HRQoL

	Model 1			Model 2			Model 3			Model 4		
	Unadjusted			Adjusted			Adjusted			Adjusted		
				Dichotomous type D personality at each time point			Dichotomous type D personality at baseline only			Continuous NA and SI scores at each time point		
	*β*	95% CI	*p* value	*β*	95% CI	*p* value	*β*	95% CI	*p* value	β	95% CI	*p* value
Time compared to baseline												
1 month	0.067	[0.042, 0.092]	< 0.001	0.058	[0.034, 0.083]	< 0.001	0.057	[0.033, 0.082]	< 0.001	0.057	[0.032, 0.081]	< 0.001
6 months	0.076	[0.050, 0.102]	< 0.001	0.056	[0.028, 0.083]	< 0.001	0.055	[0.028, 0.082]	< 0.001	0.055	[0.027, 0.082]	< 0.001
12 months	0.074	[0.046, 0.102]	< 0.001	0.068	[0.041, 0.096]	< 0.001	0.063	[0.036, 0.091]	< 0.001	0.068	[0.041, 0.096]	< 0.001
Type D personality at each time point	− 0.080	[− 0.148, − 0.012]	0.021	− 0.043	[− 0.070, − 0.017]	0.001						
Type D personality at baseline only	− 0.083	[− 0.151, − 0.014]	0.018				− 0.022	[− 0.059, 0.015]	0.24			
NA *z* score	− 0.038	[− 0.063, − 0.013]	0.003							− 0.023	[− 0.039, − 0.007]	0.004
SI *z* score	− 0.021	[− 0.046, 0.004]	0.10							− 0.006	[− 0.021, 0.009]	0.43
Interaction term (NA by SI) *z* score	− 0.001	[− 0.013, 0.011]	0.88							− 0.001	[− 0.011, 0.010]	0.91

Regression coefficient β indicates that for a unit increase in the specified predictor variable, the outcome variable Euroqol 5 dimensions questionnaire (EQ-5D) Index (scale from 0 to 1) changes with the regression coefficient

Models 2, 3, and 4 were adjusted for depression over time and age, gender, civil status, heart failure, chronic obstructive pulmonary disease, and diabetes at baseline

Additional significant patient characteristics:

- Model 1 NA by SI *z* score: NA *z* score (*β* − 0.038 [− 0.054, − 0.022])

- Model 1 NA by SI *z* score fixed: NA *z* score (*β* − 0.040 [− 0.059, − 0.022])

- Model 2: Depression *(β* − 0.186 [− 0.247, − 0.126]). Gender (*β* − 0.051 [− 0.099, − 0.002])

- Model 3: Depression (*β* − 0.179 [− 0.240, − 0.119]). Gender (*β* − 0.056 [− 0.105, − 0.007])

Chronic obstructive pulmonary disease (*β* − 0.043 [− 0.079, − 0.007])

- Model 4: Depression (*β* − 0.229 [− 0.307, − 0.150]), which also included the significant interaction term NA*depression (*β* 0.060 [0.013, 0.108])

Chronic obstructive pulmonary disease (*β* − 0.036 [− 0.072, − 0.001])

*β* regression coefficient, *CI* confidence interval, *NA* negative affectivity, *SI* social inhibition

In unadjusted analyses, dichotomized type D personality was significantly associated with impaired HRQoL at all time points (regression coefficient *β* − 0.080 [CI − 0.148, − 0.012]) (Table 5, model 1). This pattern persisted after adjustment for patient characteristics (*β* − 0.043 [CI − 0.070, − 0.017]) (Table 5, model 2). Type D personality at baseline was not significantly associated with impaired HRQoL in the multivariate model (*β* − 0.022 [C: − 0.059, 0.015]) (Table 5, model 3). This indicates that a single preoperative type D measurement is not predictive for postoperative HRQoL.

Regarding the two separate components of the type D personality construct, the continuous NA score, but not the SI score, showed a significant association with impaired HRQoL in univariate (*β* − 0.038 [CI: − 0.063, − 0.013]) and multivariate GEE models (*β* − 0.023 [CI − 0.039, − 0.007]) (Table 5, model 4). Similarly, only the continuous NA score at baseline was a significant predictor of impaired HRQoL in univariate (*β* − 0.044 [CI − 0.069, − 0.020]) and multivariate GEE models (*β* − 0.027 [CI − 0.042, − 0.011]). The interaction term NA by SI was not significantly associated with HRQoL.

At all-time points, the presence of depression as assessed by the PHQ-9 was significantly associated with impaired HRQoL in all GEE models (Table 5, additional significant patient characteristics). The aforementioned association of type D personality constructs and HRQoL persisted after removing depression from the GEE models.

## Discussion

This study shows that type D personality is exhibited in about 20% of vascular surgery patients across time, both before surgery and during 1-year postoperative follow-up. By comparison, previous studies have reported a prevalence of type D of approximately 35% in peripheral artery disease patients [10, 12, 41]. In line with former studies, the DS14 test to assess type D personality showed good internal consistency at the individual time points [8, 22]. Type D personality is currently used in a dichotomous form, thus per definition, a person belongs to the type D personality group or to the non-type D personality group. However, we found that the ICC of the continuous NA and SI scores were higher than the Cohen’s *κ* of the dichotomous form. This has also been observed in cardiac patients [20] and in patients after myocardial infarction and is in line with the criticism concerning statistical analyses of dichotomous determination of type D personality [18, 19].

Generally, type D personality is assessed only once, since it is considered a “fixed” identity. However, the current study poses the question whether the DS14 questionnaire really measures a personality trait or rather a (temporary) state of mind. While both trait and state are considered to contribute to psychological measurements, it will be interesting to investigate what exactly causes the change in type D personality status to translate this into implications for measurement or clinical intervention. In contrast to other reports in MI patients and the Netherlands Twin Register [21, 42], we found that only 9% of the patients were classified as a type D personality at all four time points, while one third of the patients changed between type D and non-type D personalities over time. This suggests that a single (baseline) measurement of the dichotomous mode of type D personality is not sufficient to determine lifelong type D personality status. Our findings are in line with the 25% type D personality rate in cardiac surgery patients, which was only consistent in 11% before and after surgery [43]. Similarly, type D personality was not stable over time in dialysis patients [44].

Type D personality was found to be strongly related to depressive symptoms in previous studies [26, 43]. What drives the relationship between depression and type D personality, specifically the NA and SI components, is a subject of debate [45]. In our study, participants with a type D personality also displayed more depression, as assessed by the PHQ-9 depression questionnaire. The PHQ-9 is a self-report screening questionnaire designed to evaluate the presence of depressive symptoms. The common practice of reporting the percentage of patients with scores above cutoff thresholds in screening questionnaires and tools for depression as disorder prevalence substantially overestimates prevalence [46]. However, the PHQ-9 is designed to make criteria-based diagnoses of depressive disorders and it is also a reliable and valid measure of depression severity. This makes the PHQ-9 a useful clinical and research tool [33]. Of note, as for type D personality, the problem for dichotomization may be identical for depression.

This study shows that the HRQoL improved after vascular surgery at all time points, with the largest improvement at 1 month postoperatively. A preoperative classification as a type D personality was not predictive of the presence or absence of an improvement in HRQoL after surgery. Thus, the DS14 personality questionnaire seems to not support decision-making when considering treatment for vascular surgery patients. However, patients with a so-called type D personality over different time points had a lower HRQoL as compared to those with a non-type D personality. This finding is in accordance with previous studies in peripheral artery disease patients showing that type D personality independently predicted impaired HRQoL more than, for example, the ankle brachial index [10, 41]. Type D personality has also been identified as an independent predictor of impaired HRQoL for other cardiovascular diseases, such as patients with coronary heart disease [47] or chronic heart failure [11] and in heart transplant recipients [48]. In line with previous studies in cardiovascular patients [6, 25], we identified that the NA component of type D personality as well as depression were significantly associated with impaired HRQoL. Consequently, screening for and treatment of depressive symptoms may provide an opportunity to improve the overall HRQoL in vascular surgery patients [49].

This study has limitations that should be addressed. First, the heterogeneous study population of patients undergoing vascular surgery precludes the possibility of drawing conclusions on treatment effectiveness for occlusive or aneurysmal disease. However, it was not the aim of this study to investigate the effect of a specific treatment on HRQoL, but rather to assess the influence of changing psychological factors on subjective health status. Second, 12 months of follow-up may be relatively short. However, the major differences in outcome variables were noted between preoperative assessment and 1 month postoperatively, with only minor changes thereafter. Finally, not all participants filled out all questionnaires at each follow-up moment. However, the lowest response rate in our study was 78% and apart from gender, marital state, and smoking status, no differences in the reported parameters were found between responders at all follow-up moments and those who did not complete one or more follow-ups. Moreover, analyses of the change in HRQoL over time as well as its association with dichotomous type D personality and its continuous scores were performed with GEE modeling which is a suitable method to accommodate for missing values.

In conclusion, this study shows that despite a point prevalence of type D personality of about 20% among vascular surgery patients, the type D personality in its current dichotomous form is not a stable trait over time. Preoperative assessment of type D personality does not predict the presence or absence of an improvement in HRQoL after a vascular surgical treatment. However, this study revealed associations between the NA component of the type D personality construct, depression and lower HRQoL. This indicates that measures of overall negative affect should be taken into account when assessing HRQoL patient-reported outcomes in vascular surgery patients.

## References

[1] Nehler MR, McDermott MM, Treat-Jacobson D, Chetter I, Regensteiner JG (2003). Functional outcomes and quality of life in peripheral arterial disease: current status. Vasc Med.

[2] Regensteiner JG, Hiatt WR, Coll JR, Criqui MH, Treat-Jacobsn D, McDermott MM, Hirsch AT (2008). The impact of peripheral arterial disease on health-related quality of life in the peripheral arterial disease awareness, risk, and treatment: new resources for survival (PARTNERS) program. Vasc Med.

[3] Liles DR, Kallen MA, Petersen LA, Bush RL (2006). Quality of life and peripheral arterial disease. J Surg Res.

[4] Hinterseher I, Kuffner H, Berth H, Gäbel G, Bötticher G, Saeger HD, Smelser D (2013). Long-term quality of life of abdominal aortic aneurysm patients under surveillance or after operative treatment. Ann Vasc Surg.

[5] Coughlin PA, Jackson D, White AD, Bailey MA, Farrow C, Scott DJA, Howell SJ (2013). Meta-analysis of prospective trials determining the short- and mid-term effect of elective open and endovascular repair of abdominal aortic aneurysms on quality of life. Br J Surg.

[6] Rumsfeld JS, Alexander KP, Goff DC (2013). Cardiovascular health: the importance of measuring patient-reported health status: a scientific statement from the American Heart Association. Circulation.

[7] Hicks CW, Lum YW (2015). Patient-reported outcome measures in vascular surgery. Semin Vasc Surg.

[8] Denollet J (2005). DS14: standard assessment of negative affectivity, social inhibition, and type D personality. Psychosom Med.

[9] Versteeg H, Spek V, Pedersen SS, Denollet J (2012). Type D personality and health status in cardiovascular disease populations: a meta-analysis of prospective studies. Eur J Prev Cardiol.

[10] Aquarius AE, Denollet J, de Vries J, Hamming JF (2007). Poor health-related quality of life in patients with peripheral arterial disease: type D personality and severity of peripheral arterial disease as independent predictors. J Vasc Surg.

[11] Schiffer AA, Pedersen SS, Widdershoven JW, Hendriks EH, Winter JB, Denollet J (2005). The distressed (type D) personality is independently associated with impaired health status and increased depressive symptoms in chronic heart failure. Eur J Cardiovasc Prev Rehabil.

[12] Aquarius AE, Smolderen KG, Hamming JF, de Vries J, Vriens PW, Denollet J (2009). Type D personality and mortality in peripheral arterial disease: a pilot study. Arch Surg.

[13] O'Dell KR, Masters KS, Spielmans GI, Maisto SA (2011). Does type-D personality predict outcomes among patients with cardiovascular disease? A meta-analytic review. J Psychosom Res.

[14] Torrent DJ, Maness MR, Capps TC, Sears SF, Whited AL, Yamaguchi DJ, Parker FM, Stoner MC (2014). Impact of psychological factors on objective ambulatory measures in patients with intermittent claudication. J Vasc Surg.

[15] Piepoli MF, Hoes AW, Agewall S (2016). European guidelines on cardiovascular disease prevention in clinical practice: the sixth joint task force of the European Society of Cardiology and Other Societies on cardiovascular disease prevention in clinical practice (constituted by representatives of 10 societies and by invited experts) developed with the special contribution of the European Association for Cardiovascular Prevention & rehabilitation (EACPR). Eur Heart J.

[16] Grande G, Romppel M, Barth J (2012). Association between type D personality and prognosis in patients with cardiovascular diseases: a systematic review and meta-analysis. Ann Behav Med.

[17] Grande G, Romppel M, Vesper JM, Schubmann R, Glaesmer H, Herrmann-Lingen C (2011). Type D personality and all-cause mortality in cardiac patients—data from a German cohort study. Psychosom Med.

[18] Ferguson E, Williams L, O'Connor RC (2009). A taxometric analysis of type-D personality. Psychosom Med.

[19] Smith TW (2011). Toward a more systematic, cumulative, and applicable science of personality and health: lessons from type D personality. Psychosom Med.

[20] Romppel M, Herrmann-Lingen C, Vesper JM, Grande G (2012). Six year stability of type-D personality in a German cohort of cardiac patients. J Psychosom Res.

[21] Kupper N, Boomsma DI, de Geus EJ, Denollet J, Willemsen G (2011). Nine-year stability of type D personality: contributions of genes and environment. Psychosom Med.

[22] Conden E, Rosenblad A, Ekselius L, Aslund C (2014). Prevalence of type D personality and factorial and temporal stability of the DS14 after myocardial infarction in a Swedish population. Scand J Psychol.

[23] Pedersen SS, Tekle FB, Hoogwegt MT, Jordaens L, Theuns DA (2012). Shock and patient preimplantation type D personality are associated with poor health status in patients with implantable cardioverter-defibrillator. Circ Cardiovasc Qual Outcomes.

[24] Kupper N, Pedersen SS, Hofer S (2013). Cross-cultural analysis of type D (distressed) personality in 6222 patients with ischemic heart disease: a study from the international HeartQoL project. Int J Cardiol.

[25] Williams L, O'Connor RC, Grubb NR, O'Carroll RE (2012). Type D personality and three-month psychosocial outcomes among patients post-myocardial infarction. J Psychosom Res.

[26] Kuijpers PMJC, Denollet J, Wellens HJJ, Crijns HM, Honig A (2007). Noncardiac chest pain in the emergency department: the role of cardiac history, anxiety or depression and type D personality. Eur J Cardiovacs Prev Rehabil.

[27] Emons WH, Meijer RR, Denollet J (2007). Negative affectivity and social inhibition in cardiovascular disease: evaluating type-D personality and its assessment using item response theory. J Psychosom Res.

[28] MacCallum RC, Zhang S, Preacher KJ, Rucker DD (2002). On the practice of dichotomization of quantitative variables. Psychol Methods.

[29] EuroQol G (1990). EuroQol—a new facility for the measurement of health-related quality of life. Health Policy.

[30] Lamers LM, Bouwmans CA, van Straten A, Donker MC, Hakkaart L (2006). Comparison of EQ-5D and SF-6D utilities in mental health patients. Health Econ.

[31] Dyer MTD, Goldsmith KA, Sharples LS, Buxton MJ (2010). A review of health utilities using the EQ-5D in studies of cardiovascular disease. Health Qual Life Out.

[32] Spitzer RL, Williams JB, Kroenke K, Linzer M, deGruy FV, Hahn SR, Brody D, Johnson JG (1994). Utility of a new procedure for diagnosing mental disorders in primary care. The PRIME-MD 1000 study. JAMA.

[33] Kroenke K, Spitzer RL, Williams JB (2001). The PHQ-9: validity of a brief depression severity measure. J Gen Intern Med.

[34] Gilbody S, Richards D, Brealey S, Hewitt C (2007). Screening for depression in medical settings with the patient health questionnaire (PHQ): a diagnostic meta-analysis. J Gen Intern Med.

[35] Wittkampf KA, Naeije L, Schene AH, Huyser J, van Weert HC (2007). Diagnostic accuracy of the mood module of the patient health questionnaire: a systematic review. Gen Hosp Psychiatry.

[36] Fayers P, David M. Quality of life—the assessment, analysis and reporting of patient-reported outcomes. John Wiley & Sons; 2016. p. 139.

[37] Twisk JW (2004). Longitudinal data analysis. A comparison between generalized estimating equations and random coefficient analysis. Eur J Epidemiol.

[38] Lewis EF (2012). Still at the drawing board improving quality of life in heart failure. Circ-Heart Fail.

[39] Solli O, Stavem K, Kristiansen IS (2010). Health-related quality of life in diabetes: the associations of complications with EQ-5D scores. Health Qual Life Outcomes.

[40] Niesink A, Trappenburg JCA, Oene GHDWV (2007). Systematic review of the effects of chronic disease management on quality-of-life in people with chronic obstructive pulmonary disease. Resp Med.

[41] Aquarius AE, Denollet J, Hamming JF, De Vries J (2005). Role of disease status and type D personality in outcomes in patients with peripheral arterial disease. Am J Cardiol.

[42] Martens EJ, Kupper N, Pedersen SS, Aquarius AE, Denollet J (2007). Type-D personality is a stable taxonomy in post-MI patients over an 18-month period. J Psychosom Res.

[43] Dannemann S, Matschke K, Einsle F, Smucker MR, Zimmermann K, Joraschky P, Weidner K, Köllner V (2010). Is type-D a stable construct? An examination of type-D personality in patients before and after cardiac surgery. J Psychosom Res.

[44] Loosman WL, de Jong RW, Haverkamp GLG, van den Beukel TO, Dekker FW, Siegert CEH, Honig A (2018). The stability of type D personality in dialysis patients. Int J Behav Med.

[45] Coyne JC, Jaarsma T, Luttik ML, van Sonderen E, van Veldhuisen DJ, Sanderman R (2011). Lack of prognostic value of type D personality for mortality in a large sample of heart failure patients. Psychosom Med.

[46] Thombs BD, Kwakkenbos L, Levis AW, Benedetti A (2018). Addressing overestimation of the prevalence of depression based on self-report screening questionnaires. CMAJ.

[47] Denollet J, Vaes J, Brutsaert DL (2000). Inadequate response to treatment in coronary heart disease: adverse effects of type D personality and younger age on 5-year prognosis and quality of life. Circulation.

[48] Pedersen SS, Holkamp PG, Caliskan K, van Domburg RT, Erdman RAM, Balk AHMM (2006). Type D personality is associated with impaired health-related quality of life 7 years following heart transplantation. J Psychosom Res.

[49] Smith SC, Benjamin EJ, Bonow RO (2011). AHA/ACCF secondary prevention and risk reduction therapy for patients with coronary and other atherosclerotic vascular disease: 2011 update: a guideline from the American Heart Association and American College of Cardiology Foundation endorsed by the World Heart Federation and the Preventive Cardiovascular Nurses Association. J Am Coll Cardiol.

